# Pure Camphor and a Thujone-Camphor Mixture as Eco-Friendly Antifeedants against Larvae and Adults of the Colorado Potato Beetle

**DOI:** 10.3390/plants11243587

**Published:** 2022-12-19

**Authors:** Jelica Lazarević, Igor Kostić, Darka Šešlija Jovanović, Dušica Ćalić, Slobodan Milanović, Miroslav Kostić

**Affiliations:** 1 Institute for Biological Research “Siniša Stanković”—National Institute of the Republic of Serbia, University of Belgrade, Bulevar Despota Stefana 142, 11060 Belgrade, Serbia; 2 Institute for Multidisciplinary Research, University of Belgrade, Kneza Višeslava 1, 11030 Belgrade, Serbia; 3 Faculty of Forestry, University of Belgrade, Kneza Višeslava 1, 11030 Belgrade, Serbia; 4 Department of Forest Protection and Wildlife Management, Faculty of Forestry and Wood Technology, Mendel University, Zemědělská 3, 61300 Brno, Czech Republic; 5 Institute for Medicinal Plant Research “Dr Josif Pančić”, Tadeuša Košćuška 1, 11000 Belgrade, Serbia

**Keywords:** *Leptinotarsa decemlineata*, botanicals, monoterpene ketone, deterrent, repellent, irritant

## Abstract

The Colorado potato beetle (CPB) is a serious pest of economically important Solanaceae species. The use of essential oil compounds in pest management has been proposed as an alternative to harmful chemical insecticides that disturb human health and ecosystem functioning. We examined the antifeedant activity of three concentrations (0.125%, 0.25% and 0.5%) of pure camphor and a thujone-camphor mixture against 3rd instar larvae and adults. Their efficacy was evaluated according to the degree of leaf damage and avoidance of treated leaves by the CPB. Treatment of potato leaves significantly reduced leaf damage compared to the control. Leaf protection increased at higher concentrations of the examined compounds. Camphor was more effective against larvae and the thujone-camphor mixture was more effective against adults. Additionally, adults moved faster towards the control leaf disc in the two-choice olfactometer assay if an alternative disc was treated with a thujone-camphor mixture, whereas larvae responded similarly to the two potential repellents. However, after contact with the leaf disc treated with the highest compound concentration, the larvae escaped faster from the thujone-camphor mixture than from pure camphor. In conclusion, both examined compounds are promising eco-friendly antifeedants, but their efficacy depends on the developmental stage of the beetle, compound type and applied concentration.

## 1. Introduction

Since the 1940s, efforts against crop pests have mainly relied on the application of chemical insecticides. These have significantly reduced crop loss. However, it was soon recognized that these insecticides compromise ecosystem functioning and human health due to the prolonged persistence of their residues as well as their low specificity [1,2,3]. Additionally, many insect pests developed resistance to these simple pesticidal molecules [4,5]. During the search for alternative means of pest control, it was proposed that plant protection could be based on natural mechanisms of plant defence. Thus, plants attacked by herbivores emit volatiles (terpenoids, phenylpropanoids and others), which induce avoidance behaviour in pests and/or attract their natural enemies [6]. Use of these volatiles as natural defence compounds can be of great importance in integrated pest management as they can mitigate many of the disadvantages described for chemical insecticides. Various plant extracts, essential oils (EOs), EO fractions and EO compounds were tested for their activity against pests (reviewed in [7,8,9,10,11,12,13]). In addition to the toxic effects of plant-derived compounds, other activities relevant to pest control, such as reduced locomotion, decreased growth and progeny production, repellence and feeding/oviposition deterrence have been recorded at sublethal concentrations [14,15,16,17,18,19,20]. However, using plant-derived compounds in pest control has several drawbacks that challenge their commercial application. The main shortcomings are their low persistence, low efficacy in the field, high cost of production and unpredictable supply of high-quality pesticidal plants due to their sensitivity to environmental variations [12,21,22]. Currently, the commercial development of active substances also considers the natural and artificial blends of terpenoids, which underscores the importance of pest behavioural modification and appropriate nanotechnology-based formulations for the overall efficacy of compounds [23].

Behavioural modification of pest insects exposed to terpenoids and other secondary metabolites can effectively reduce or even prevent host-plant acceptance for feeding, leading to death from starvation, a decrease in population growth and reduced crop losses [9]. Volatile compounds act through olfactory and/or taste receptors, forcing insects to move away from the plant (repellent), inhibiting initiation or continuation of feeding (suppressant and deterrent effects, respectively) [24]. Repellents, suppressants and deterrents are involved in the antifeedant activity. Spraying with plant chemicals masks the volatiles of host plants and interferes with host finding by pests. Even if contact with chemical residues on host plants occurs, pests are irritated and stimulated to escape from the source of irritation [25,26]. Studies on many pest insects have revealed the feeding deterrent, odour masking and escape behavioural responses to EOs and EO compounds (reviewed in [11,27,28,29,30]). Herein, we used the Colorado potato beetle (CPB), *Leptinotarsa decemlineata* (Say) (Coleoptera: Chrysomelidae), to study the behavioural modifications in response to natural compounds.

CPB is a serious pest of solanaceous plants. Its larvae and adults feed on leaves and cause significant damage to many hosts, including potato *Solanum tuberosum* L., tomato *Solanum lycopersicum* L., eggplant *Solanum melongena* L. and others [31]. After long-term application of synthetic chemicals, CPB developed resistance to a large number of insecticides, thereby increasing the cost of potato production [32]. An alternative control measure is a push-pull strategy, which involves different chemical cues that either repel (e.g., antifeedants) or attract (e.g., host plant volatiles) insect pests [33]. The composition of volatiles emitted from intact and CPB-infested potato plants is known, and various blends of synthetic and natural compounds or individual chemicals have been tested in the laboratory for their effect on oriented CPB movements and antifeeding activity [34,35,36,37,38,39,40,41,42]. It was reported that crude non-host plant extracts, EOs and their terpenoid compounds are potent CPB antifeedants [43,44,45,46,47,48,49]. In the present paper, pure camphor and a thujone-camphor mixture were evaluated for their antifeedant activity against CPB.

Camphor and thujone are bicyclic oxygenated monoterpene ketones with the chemical formula C_10_H_16_O. Most of their physicochemical characteristics are similar, although camphor is slightly more lipophilic and thujone has a slightly higher surface tension and almost 3-fold higher dreiding energy [50]. The camphor and thujone contents in EOs vary depending on the plant species and chemotype, plant organ and development stage, as well as on the environmental conditions related to the specific locality and season. The highest camphor content was found in *Cinnamomum camphora* (L.) J. Presl where it can reach values above 80% [51]. Also, camphor was detected as the major compound in EOs isolated from some chemotypes of *Ocimum basilicum* L. [52], *Artemisia sieberi* Besser [53], *Achillea sieheana* Stapf [54], *Tanacetum parthenium* L. [55] and *Lavandula cariensis* Boiss. [56]. Thujone-rich EOs have been isolated from *Artemisia absinthium* L. [57], *T. argyrophyllum* (C. Koch) Tvzel. var. *argyrophyllum*, *T. praeterium* (Horwood) Heywood subsp. *massicyticum* [58], *T. vulgare* L., *Thuja occidentalis* L. [59,60] and *Senecio chrysanthemoides* D. C. [61]. Sage, *Salvia officinalis* L. EO (SEO), contains both thujone and camphor. In Europe, depending on the locality, the contents of α-thujone, β-thujone and camphor in SEO are in the range of 3.0–26.6%, 1.5–12.9% and 11.3–29.8%, respectively [62]. These ketone compounds and the EOs that contain them exert considerable effects on pest insect survival, reproduction, behaviour and physiology [40,63,64,65,66,67,68,69,70,71,72,73,74,75,76,77,78,79,80,81,82,83,84,85].

In our previous study, we showed that pure camphor and the thujone-rich fraction of SEO possessed a good feeding deterrent activity against the larvae and adults of *L. decemlineata* [40]. After 96 h of larval exposure to 0.5% camphor and adult exposure to 0.5% thujone-rich SEO fraction, leaf damage was lower than the damage of leaves treated with sage oil. Herein we mixed thujone and camphor at a ratio similar to the effective SEO fraction and examined the influence of the three concentrations (0.125%, 0.25% and 0.5%) of pure camphor and thujone-camphor mixture on leaf damage by 3rd instar larvae and adults, as well as larval and adult movements away from the odour stimulus in an olfactometer. An additional experiment was carried out to examine larval behaviour after contact with the test compounds. Thus, our overall aim was to detect which compound(s) provide better protection to potato leaves because of impaired pest feeding and host finding, as well as through contact irritancy.

## 2. Results

### 2.1. Feeding Deterrent Effects of Pure Camphor and the Thujone-Camphor Mixture

**Figure 1:**
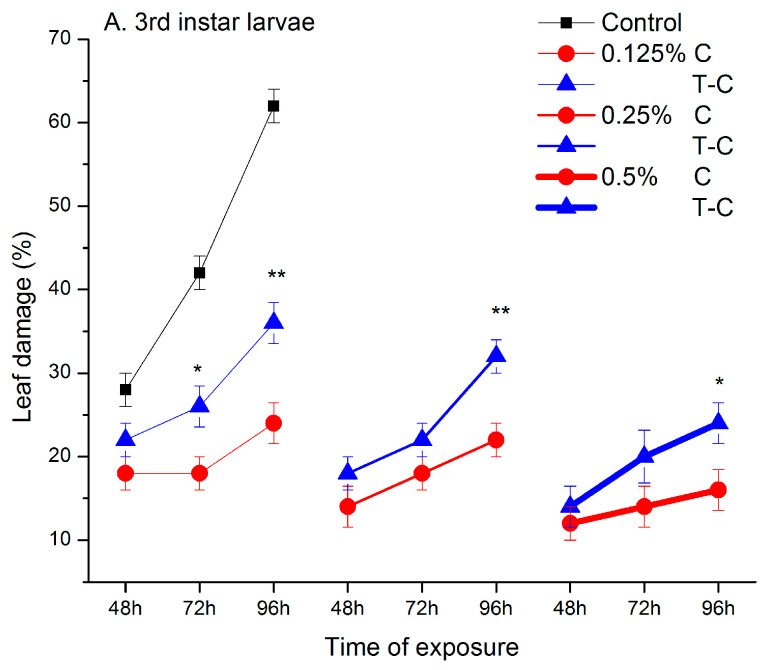

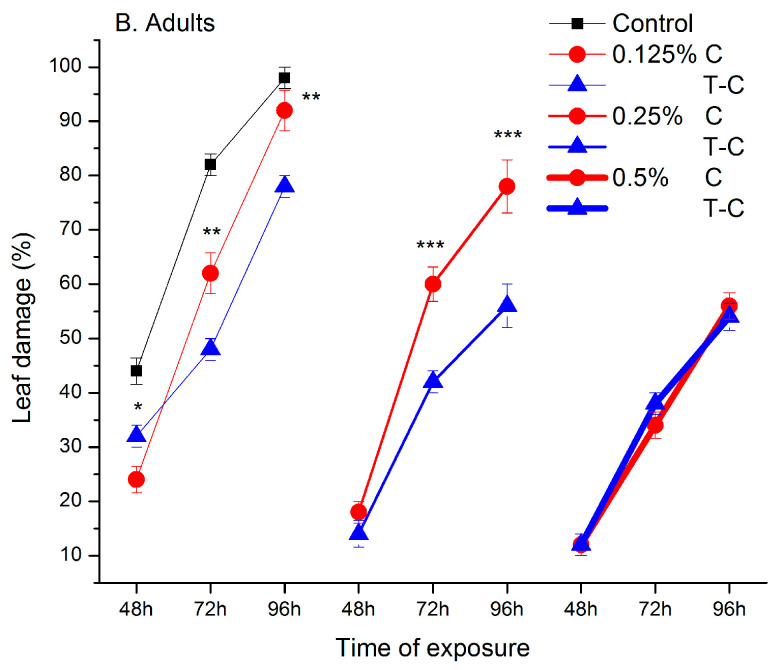
Percentage of leaf damage by CPB larvae (**A**) and adults (**B**) exposed to different concentrations of pure camphor (C, red lines) and the thujone-camphor mixture (T-C, blue lines). Asterisks indicate significant differences between C and T-C (LSM contrasts, * *p* < 0.05, ** *p* < 0.01, *** *p* < 0.001).

The consumption of ketone treated leaves by 3rd instar larvae was significantly reduced after 48 h (F_6,28_ = 6.71, *p* < 0.001), 72 h (F_6,28_ = 15.68, *p* < 0.001) and 96 h (F_6,28_ = 45.07, *p* < 0.001) (Figure 1A). Post hoc comparisons revealed significant differences (*p* < 0.001) from the control in all treatment groups, except in larvae exposed to the lowest concentration of the camphor-thujone mixture for 48 h (*p* = 0.2247). Also, leaf damage caused by adult consumption was significantly decreased (48 h: F_6,28_ = 29.76, *p* < 0.001; 72 h: F_6,28_ = 43.10, *p* < 0.001; 96 h: F_6,28_ = 31.14, *p* < 0.001) (Figure 1B). Only adults exposed to the lowest concentration of camphor for 96 h did not differ from the control (*p* = 0.615).

Analysis of the between-subject effects in repeated measures two-way ANOVA showed that leaf damage was significantly affected by the monoterpene ketone(s) type and their concentration both in larvae and adults (significant K and C effects in Table 1). On average, pure camphor provided better protection against CPB larvae than the ketone mixture (grand means for C/T-C: 17.33/23.78%) with significant differences recorded at the end of observation (96 h) (Figure 1A). The thujone-camphor mixture was more efficient against adults (grand means for C/T-C: 48.44/41.56%), however, at the highest concentration both ketone formulations were equally effective (Figure 1B).

Leaf damage gradually decreased from the lowest to the highest concentration. On average, a comparison between the lowest and highest concentrations revealed 30% and 40% decreases in leaf damage caused by larvae and adults, respectively. The pattern of the decrease in leaf damage was similar in larvae exposed to pure camphor or thujone-camphor. In contrast, significant K × C interaction was recorded in adults (Table 1). At medium concentration (0.25%) adults exposed to the ketone mixture caused a significant reduction in leaf damage relative to the lowest concentration (0.125%) (*p* = 0.001), whereas leaf consumption by camphor-exposed adults showed no differences between these groups (*p* = 0.206).

Leaf consumption was significantly increased during the time of exposure to ketones both by larvae and adults (Figure 1, significant T effect in Table 1). Analysis of within-subject effects revealed that the shape of the response curves differed between ketone(s) (significant T × K interaction in Table 1). Namely, in larvae the increase in leaf damage observed from 48 to 72 h was significant for the ketone mixture (*p* = 0.004) and non-significant for pure camphor (*p* = 0.562). In adults, the leaf damage increase was significant between 48 h and 72 h and between 72 and 96 h, but the increase slope was steeper for pure camphor (Figure 1). While time response curves were parallel for the different concentrations in larvae (non-significant T × C interaction term in Table 1), in adults the curves became steeper at higher concentrations (Figure 1, significant T × C interaction term in Table 1).

**Figure 2:**
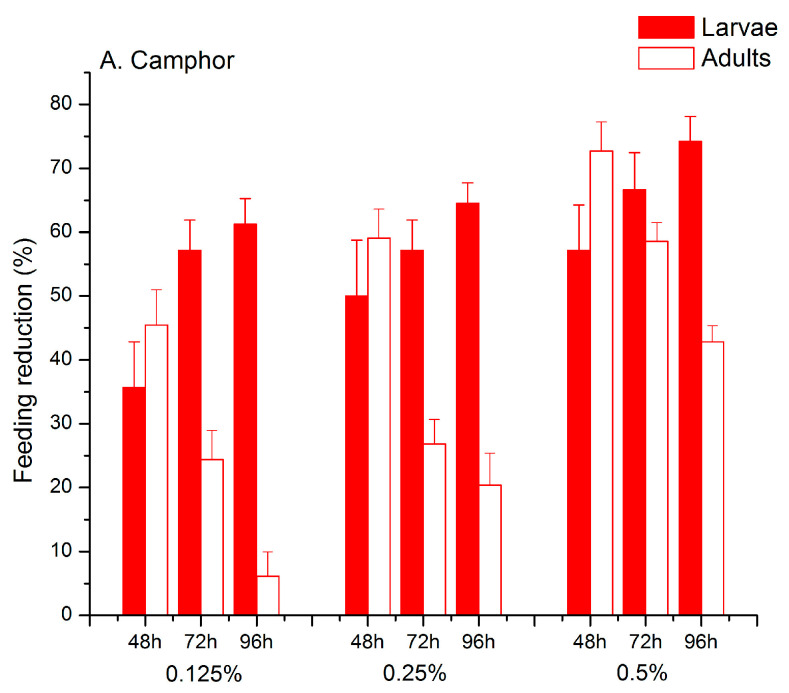

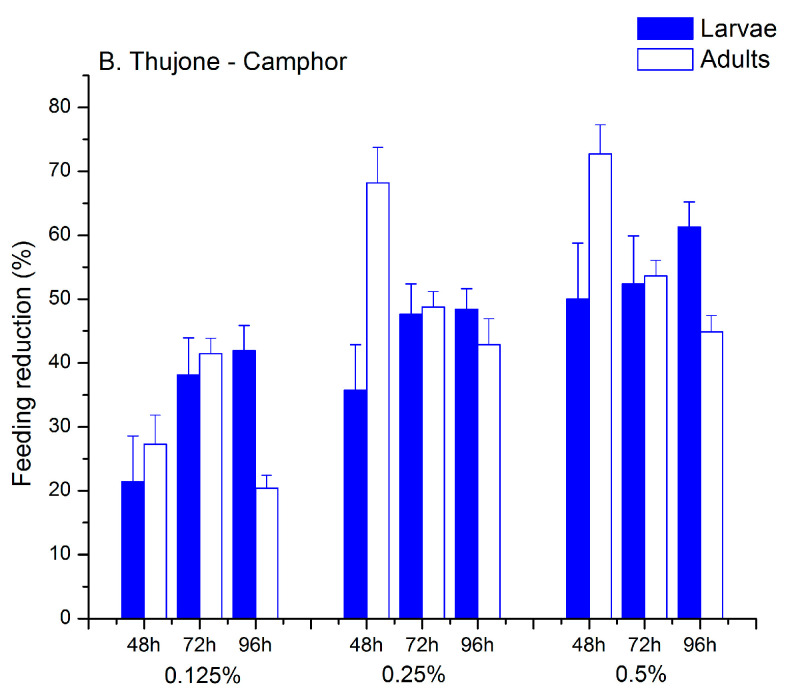
Feeding reduction index in CPB larvae and adults exposed to pure camphor (**A**) and the thujone-camphor mixture (**B**) at three different concentrations.

The percentage of feeding reduction relative to the control (FI, feeding reduction index) differed between larvae and adults (Figure 2; significant D effect in Table 2). Larvae were more sensitive than adults to the ketone leaf treatments, i.e., leaf damage was lower in the larvae sample than in the adult sample (grand mean for larvae/adults: 51.15/43.15%). Larvae were significantly more sensitive to pure camphor than the thujone-camphor mixture (*p* = 0.001). A similar degree of leaf damage reduction was recorded in adults exposed to C and T-C (*p* = 0.158) (significant D × K interaction in Table 2). Both camphor and the thujone-camphor mixture provoked an increase in feeding reduction during the observation time in larvae and decreased feeding reduction in adults (Figure 2, significant T × D interaction in Table 2). The slope of feeding reduction increase observed in larvae did not differ between C and T-C, whereas in adults a steeper decrease in feeding reduction was obtained for the camphor than for the thujone-camphor mixture (Figure 2, significant T × D × K interaction in Table 2).

### 2.2. Behavioural Responses in the Absence of Contact and after Contact with Ketone Treated Leaves

Both larvae and adults needed more time to choose the control leaf disc in the olfactometer when an alternative disc was treated with ketones than when both discs were treated with the solvent (ethanol) (Table 3). Pure camphor and the thujone-camphor mixture were equally effective in confusing 3rd instar larvae and exhibited similar trends of choice time change with concentration (non-significant K and K × C effects in Table 4). However, in adults, camphor induced a longer choice time than the ketone mixture (significant K effect in Table 4). On average, the choice time differed between the developmental stages (significant D effect in Table 5), but the only significant difference between larvae and adults was recorded for the 0.25% thujone-camphor mixture (*p* = 0.048).

On average, the ketone formulation did not influence the speed of movement away from the treated leaf disc (non-significant K effect for larval escape time in Table 4). However, a posteriori comparison revealed that the larvae were faster in escaping camphor than the thujone-camphor treated disc at a concentration of 0.1% (*p* = 0.010), whereas at 0.5%, the thujone-camphor mixture was more irritant (*p* < 0.001). The escape time significantly decreased with concentration, but the decrease was steeper in the thujone-camphor group (significant K and K × C terms in Table 4). In the camphor group, the concentration did not have a significant effect on larval movements (0.1 vs. 0.25%: *p* = 0.098; 0.1 vs. 0.5%: *p* = 0.138; 0.25 vs. 0.5%: *p* = 0.860), whereas in the thujone-camphor group all differences were significant (0.1 vs. 0.25%: *p* = 0.003; 0.1 vs. 0.5%: *p* < 0.001; 0.25 vs. 0.5%: *p* < 0.001).

## 3. Discussion

The use of semiochemicals that affect pest host finding, feeding, mating and oviposition is considered as one of the safest means of pest control [33,41]. Behavioral modifications with significant implications for pest population dynamics occur at sublethal concentrations of chemicals, thus lowering the risk of their harmful effects on human health and the environment. Plant-derived compounds can be an eco-friendly replacement for synthetic repellents and their effects on insect behaviour are studied by applying different research methods [27,28]. In the present study, we used three methods to evaluate the efficacy of camphor and a thujone-camphor mixture against CPB. Firstly, monitoring leaf damage during 4 days of feeding in the no-choice assay showed the level of reduction in treated leaf consumption by the CPB. During this period, we did not record any larval or adult mortality, which is consistent with previous study showing low mortality after exposure to 0.5% camphor and a thujone-rich fraction of SEO [40]. Secondly, we used the choice assay in an olfactometer to examine whether exposure of potato leaf disc to the tested compounds hindered host finding. In the third assay, we allowed contact with the treated leaf disc to assess the level of compound irritancy in CPB larvae. Our results showed that CPB exposed to monoterpene ketones on potato leaves significantly reduced leaf consumption, disturbed host finding and provoked a contact irritancy response.

It has been suggested that more oxidized compounds are suitable pest toxicants and antifeedants although many other physicochemical traits can affect compound interaction with target molecules [86,87]. For example, Zaio et al. [88] observed a positive correlation between compound repellence against *Sitophilus zeamais* (Motsch.) with lipophilicity (logP) and polar surface (PS). The camphor and thujone that were examined in the present study for antifeedant activity against CPB belong to oxygenated monoterpenes. Although they have similar logP and PS values, we recorded different effects on feeding reduction and adult choice time prolongation relative to the control. Gonzalez-Coloma et al. [89] studied feeding inhibition in CPB adults exposed to 47 samples of *Lavandula luisieri* L. EOs and reported that it strongly correlated with the combination of the two EO compounds—camphor and hydroxyketone. In addition to physicochemical characteristics of ketones, the insect response can be affected by characteristics such as the composition and thickness of the cuticle or the structure of different target molecules. A mixture of compounds can exert a response that differs from the effects of pure compounds [14].

Ketone toxicity could be a consequence of the modulation of the insect gamma-aminobutyric acid (GABA) system, increased oxidative stress, inhibition of detoxification enzymes and the impairment of digestion and transport of nutrients [9,71,82,90]. The specific behavioural response depends on the degree of compound delivery to the target receptors (olfactory and gustatory), their interaction with the sensory periphery and signal transduction to the higher brain [26]. In CPB, 26 putative odour-binding receptors were identified that are expressed in different adult tissues including antennae, head, thorax and legs [91]. Different compounds bind to different receptors and can also block the binding of host odours and feeding stimuli, leading to host avoidance, masking and feeding deterrence. For example, camphor and α-thujone triggered significant excitatory responses in one type of *Aedes aegypti* (L.) olfactory receptor neurons and an inhibitory response in another type [77]. At high concentrations, more receptors are activated, and the amplitude and duration of the response are increased [92]. In accordance with many studies on CPB (e.g., [45,93,94,95]) and other pests (e.g., [96,97,98,99]) that confirmed the concentration-dependence of behavioural responses to plant chemicals, we also observed a more intense response to higher concentrations of camphor and the thujone-camphor mixture in all three assays.

Complete inhibition of CPB feeding was detected in the no-choice test with 4th instar larvae exposed to potato leaf discs treated with *Satureja hortensis* L. and *Thymus vulgaris* L. EOs [94] and the aqueous suspension of *Tanacetum vulgare* [100]. Among the methanolic extracts of 75 examined plant species, the lowest median effective doses were found in *Angelica archangelica* L. fruits, *Grindelia camporum* Greene stem and *Inula auriculata* Boiss. and Balansa stem [101]. Feeding reduction above 90% was obtained for oxygenated sesquiterpenes [47] and neem extract [102]. The methanolic extract of *Humulus lupulus* L. significantly reduced feeding so that at the concentration of 2%, the remaining leaf amount was 4-fold higher than in the control [45]. Medium values were shown with the aqueous extract of *Artemisia absinthium* [103], whereas the aqueous extracts of *Thymus serpyllum* L. [104] and *Origanum vulgare* L. were weak deterrents [48].

Camphor- or *T. vulgare* EO-treated filter paper exhibited moderate repellence in the olfactometer assay [36]. However, when control and *Tanacetum* EO-treated leaf discs were presented as alternatives the adults and larvae always moved towards the control disc [105]. Consistent with the presented results, adults and larvae exposed to higher *Tanacetum* EO concentrations made faster decisions to move towards the control [49]. Similarly, the mixture of non-host and host plant odour impaired CPB behaviour in the olfactometer and considerably reduced the walking speed and the time spent walking towards the stimulus [37]. The thujone-rich fraction of SEO was a weaker repellent [93] but a stronger feeding deterrent than SEO for CPB adults [40]. This suggests that other components contributed to EO repellence but antagonized thujone and camphor activity.

Schearer [36] and Panasiuk [35] showed that CPB escaped from the filter paper treated with camphor and the thujone-rich *T. vulgare* EO. Also, leaves treated with α,β-thujone were avoided for 24 h, whereas an avoidance response to camphor was much less persistent [35]. We observed that CPB larvae quickly escaped from the leaves treated with the highest concentration of the thujone-camphor mixture. Treatment of potato leaves with extracts of *H. lupulus* decreased the resting time on leaves and increased the resting time on the dish and the frequency of walking both in larvae and adults [95]. Similarly, using a multiple choice test in a large Petri dish showed that adults avoided zones of potato leaves treated with the ethanol extract of *L. angustifolia*, which contained geraniol, linalool, 1,8-cineole and camphor [106].

Behavioural modulation by camphor and thujone was recorded in other pests as well. Depending on the concentration, both α-thujone and/or camphor can be repellent for *S. zeamais* [70], *Tribolium castaneum* (Herbst) and *Lasioderma serricorne* (F.) [76], *S. granarius* (L.), *Prostephanus truncatus* (Horn) [63], *Aegorhinus nodipennis* (Hope) [72] and *Drosophila melanogaster* Meigen [84]. β-thujone deterred feeding of the aphid *Myzus persicae* (Sulz.) [80], α-thujone was a feeding deterrent for an apple pest *Cydia pomonella* L. [107] and a thujone/isothujone rich fraction of *Thuja plicata* Donn EO displayed a feeding deterrent activity against the pine weevil *Pissodes strobi* Peck [108].

The activities of pure camphor and the thujone-camphor mixture depended on the developmental stage. In larvae, camphor had higher feeding deterrent and contact irritant activities than the thujone-camphor mixture, whereas both compounds were equally effective in interfering with adult movement towards the control disc in the olfactometer. In adults, the ketone mixture had a higher deterrent activity but was ineffective in the olfactometer assay, although a trend of increasing choice time was noted. The stage-specific response could reflect the differences in receptors [109] and the detoxification system [110] in the two stages. Other studies also showed that the sensitivity to plant-derived compounds differed between young vs. old larvae and larvae vs. adults. For example, α-methylenelactone in CPB larvae induced a higher deterrent effect in the no-choice assay, whereas adults were deterred to a greater extent in the choice assay [111]. This result implies that in larvae, α-methylenelactone deterred feeding through physiological toxicity, whereas in adults deterrence resulted from modified behaviour. After 48 h of exposure to potato leaves treated with the methanolic extract of *H. lupulus*, adults completely ceased feeding at a 10-fold lower concentration of extract than 3rd instar larvae [95]. Limonoid epilimonol provoked a higher reduction in leaf consumption in younger than in older CPB larvae [112]. Toxicity studies also revealed the development of stage-specific effects of terpenes in CPB. For example, compared to 2nd instar larvae, CPB adults were 10-fold more sensitive to the ethanolic extract of *S. cilicica* P. H. Davis and 5-fold less sensitive to *S. montana* L.; the young 1st instar larvae were more sensitive to *S. montana* than the older 4th instar larvae [113]. Generally, the larvae seemed to be more sensitive to oxygenated monoterpenes [114]. Our results also showed development stage-specific changes in leaf damage with time. In larvae, a less apparent damage increase was observed for the camphor than for the thujone-camphor mixture, whereas in adults, leaf damage increased more slowly with the ketone mixture. Since we monitored leaf damage for up to 4 days, the obtained results on feeding reduction could be due to not only behavioural modification but also to post-ingestive physiological toxicity. Interestingly, the feeding reduction effect of both compounds increased in larvae and decreased in adults with time. Further research is needed to elucidate which receptor or detoxification system plays a role in the opposite responses to terpenoids in larvae and adults.

The use of botanical pesticides is increasing and several formulations have been commercialized [115]. Our results point to the potential for use of both camphor and the thujone-camphor mixture as antifeedants in integrated management of CPB. Despite concerns that camphor and thujone can be toxic to humans, it has been established that the acceptable daily intake doses can be relatively high [116,117]. Another concern is related to their effects on non-target organisms for which low no-effect doses were determined e.g., [118]. It is necessary to search for appropriate formulations of botanical antifeedants that will provide high stability and efficacy. It was shown recently that the encapsulated anise EO formulation has improved stability and stronger antifeedant effects against CPB larvae [119]. An effective formulation should be further tested in the field together with studying its side effects on non-target organisms and antifeedant integration with other control strategies [120].

## 4. Materials and Methods

### 4.1. Chemicals

**Figure 3:**
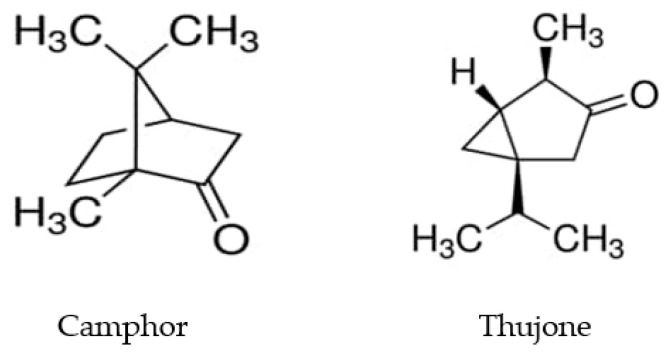
Structural formulae of camphor and thujone.

Ketones used in the present experiments were purchased from Sigma Aldrich (St. Louis, MO, USA): (±)-camphor (cat. no. 148075) and α,β-thujone (cat. no. 89230) (Figure 3). Pure camphor and the 63.5% α,β-thujone and 36.5% camphor mixture were used according to previous results on high feeding deterrence of camphor and a fraction of sage oil that contained 48.99% α-thujone, 7.16% β-thujone and 32.27% camphor [40,121].

### 4.2. Rearing Insects

CPB adult individuals were collected at the location of Dobanovci, Serbia (44° 49′ 35″ N; 20° 13′ 30″ E), from potato (*S. tuberosum*) in fields not treated with pesticides. Adults were placed in glass cylinders with potato leaves from plants grown in a glass house. The Desiree variety of potato plants aged 6–7 weeks and 25–30 cm high was used in the bioassays. After the laying of eggs on potato leaves, the adults were removed and monitoring of the successive developmental stages was initiated. Identification of CPB life stages (egg hatching, larval moulting and adult eclosion) was performed according to Boiteau and Le Blanc [122]. After moulting, larvae were transferred to clean glass cylinders. Bioassays were carried out on 3rd instar larvae 1 day after moulting and on 4-day-old adults in a microclimate chamber (Danfoss, EKH 20 operational system, Netherlands) under conditions optimal for CPB development, at 27 ± 1 °C, RH = 60 ± 5% and a 16:8 h L:D photoperiod.

### 4.3. Feeding Deterrent Activity of Monoterpene Ketones

Potato plants (cultivar Desiree) of uniform age, height and leaf mass were grown in pots and watered regularly to keep the soil moistened. In the treatment group, plants were sprayed with a TLC sprayer (Sigma-Aldrich) with the ketones diluted in ethanol as follows: 0.125%, 0.25% and 0.5%, while control plants were sprayed with 96% ethanol. For each treatment plants were sprayed with 40 mL of solution per m^2^ of the potato plant and air-dried for 15 min at room temperature. Six 3rd instar larvae or six adults (three females and three males) were starved for 24 h, placed on treated potato leaves, covered with glass cylinders and placed in a microclimate chamber. Insects were exposed to treated leaves for 48 h, 72 h and 96 h. After exposure, potato leaf damage was visually estimated using a 0–10 scale (where undamaged plants were estimated as 0 = 0% and 10 = 100% for completely consumed leaves) [49]. The bioassay was set up in five replicates per experimental group (one control group and 6 groups treated with 3 concentrations of 2 compounds). The feeding reduction index (FI) was calculated according to the formula:

FI = (C − T)/C × 100(1)

where C is the control leaf damage and T is the treated leaf damage [123].

### 4.4. CPB Behaviour in Olfactometer and Escape Bioassays

An olfactometer apparatus was designed to assess the repellent effect of monoterpene ketones in CPB larvae and adults by a two-choice method. The olfactometer was made of thick glass with dimensions of the usable space of 28 × 15 × 5 cm (length × width × height). Air was allowed to flow into two entrances, through the expanded part of the olfactometer (15 × 7 cm) and through three tunnels (16.5 × 3 cm each) towards two exits on the opposite side. The expanded part of the olfactometer prevented air turbulence. The air pump and rubber-coated tubes (9 mm in diameter) allowed inlet air to the manifold and regulated the airflow, whereas the rotameter and the glass air hub with activated charcoal served to neutralize the odoriferous substances in the air. Leaf discs were cut with a cork-borer (20 mm in diameter) and immersed for 3 s in an ethanolic solution of monoterpene ketones at concentrations of 0.125%, 0.25% and 0.5%. Control leaf discs were immersed in 96% ethanol. After air-drying for 15 min, control and treated leaf discs were placed into the right and left tunnels of the olfactometer, respectively. In the control group, leaf discs treated with ethanol were placed in both tunnels. The 3rd instar larvae and female adults were starved 24 h prior to the bioassay. In each trial, an individual was placed in the olfactometer at a distance of 21 cm from the potato leaf disc for female adults, and 2 cm for the 3rd instar larvae. The time needed for each individual to choose and move towards the control leaf disc was recorded and referred to as the ‘choice time’.

In the no-choice (contact) bioassay, each treated leaf disc was placed onto a glass Petri dish (9 × 1.5 cm) and one 3rd instar larva, previously starved for 24 h, was placed on the leaf disc. The time that a larva remained on the disc, i.e., the time before it left the disc, was monitored for 5 min and named the ‘escape time’. None of the control larvae left the leaf disc during the observation period.

Both bioassays were set for 10 individuals per experimental group.

### 4.5. Statistical Analysis

All statistical analyses were performed using the software package Statistica 7.0 (StatSoft, Inc., Tulsa, OK, USA). Data were tested for normality of distribution by the Kolmogorov Smirnov test and for homogeneity of variances by the Levene test. Assumptions were satisfied for untransformed values of leaf damage, the feeding reduction index and adult choice time in the olfactometer. Square root transformation and logarithm square root were used for larval choice and escape time, respectively.

Leaf damage caused by larvae and adults after 48 h, 72 h and 96 h was analysed by two-way repeated measures ANOVA with ketone type and ketone concentrations as between-subject factors, and the time of exposure as the within-subject (repeated) factor. Additionally, three-way repeated ANOVA was used for FI analysis with the developmental stage, ketone(s) type and concentration as between-subject factors. With this procedure we tested the significance of the effects of the main factors and their interaction with the repeated factor (time). The significant effect of a factor and its interaction with time indicated significant differences in the trait level and in the shape of trait changes during time, respectively. The lack of significant interactions with time pointed to parallel response curves [124].

Within each exposure time, one-way ANOVA and the Dunnett test were carried out to test for significant changes in the damage to camphor and thujone-camphor treated leaves relative to the control group. The same analysis was performed to compare the larval and adult choice time between the control and treatment groups. To estimate the significance of the main and interaction effects of the ketone type and concentration on the choice and escape time, two-way ANOVA was carried out. All ANOVAs were followed by Tukey’s post hoc test and planned comparisons (LSM contrasts).

## Figures and Tables

**Table 1:** Repeated measures ANOVA for the leaf damage during the exposure time in CPB larvae and adults exposed to ketone monoterpenes (pure camphor or the thujone-camphor mixture) at different concentrations. Significant effects are given in bold.

			Larvae			Adults	
Source of Variation	df	MS	F	*p*	MS	F	*p*
*Between subjects*							
Ketone (K)	1	934.4	16.49	**0.001**	1067.8	14.90	**<0.001**
Concentration (C)	2	407.8	7.20	**0.004**	3523.3	49.16	**<0.001**
K × C	2	14.4	0.25	0.777	441.1	6.16	**0.007**
Error	24	56.7			71.7		
*Within subjects*							
Time (T)	2	671.1	61.95	**<0.001**	19,123.3	849.93	**<0.001**
T × K	2	84.4	7.79	**0.001**	401.1	17.83	**<0.001**
T × C	4	21.1	1.95	0.118	226.7	10.07	**<0.001**
T × K × C	4	4.4	0.41	0.800	124.4	5.53	**0.001**
Error	48	10.8			22.5		

**Table 2:** Repeated measures ANOVA of the feeding reduction index during 48–96 h of exposure of CPB larvae and adults to different concentrations of camphor and the thujone-camphor mixture. Significant effects are given in bold.

Source of Variation	df	MS	F	*p*
*Between-subjects*				
Dev. stage (D)	1	2879.9	11.57	**0.001**
Ketone (K)	1	553.5	2.22	0.143
Concentration (C)	2	8546.3	34.33	**<0.001**
D × K	1	5055.6	20.31	**<0.001**
D × C	2	610.1	2.45	0.097
K × C	2	374.6	1.51	0.232
D × K × C	2	401.2	1.61	0.210
Error	48	248.9		
*Within-subjects*				
Time (T)	2	472.8	7.19	**0.001**
T × D	2	7661.1	116.49	**<0.001**
T × K	2	176.5	2.68	0.073
T × C	4	365.3	5.55	**0.001**
T × D × K	2	437.9	6.66	**0.002**
T × D × C	4	88.8	1.35	0.257
T × K × C	4	161.5	2.46	0.051
T × D × K × C	4	141.5	2.15	0.080
Error	96	65.8		

**Table 3:** The time needed for the insects to choose and move towards the control leaf disc in the olfactometer and the time needed to escape after contact with the treated leaf disc depending on the ketone type and concentration. F, *p* values were obtained from one-way ANOVA. Significant differences from the control group are marked in bold (Dunnett test, *p* < 0.05).

		Larval Choice Time		Adult Choice Time		Larval Escape Time	
Ketone	Conc. (%)	Mean	SE	Mean	SE	Mean	SE
Camphor	0.125	**199.8**	**13.42**	**195.6**	**26.15**	96.9	9.67
	0.25	**193.5**	**20.03**	**182.3**	**38.73**	74.5	14.92
	0.5	115.7	20.54	126.0	29.35	71.2	12.53
Thujone-camphor	0.125	**196.3**	**22.38**	143.0	25.88	241.6	57.03
	0.25	**187.3**	**22.31**	127.0	26.20	89.4	11.99
	0.5	106.7	6.67	88.0	22.33	18.9	2.58
Control	0	70.9	10.75	73.0	14.08		
	ANOVA	F_6,63_ = 10.30 *p* < 0.001		F_6,63_ = 2.78 *p* = 0.018		F_5,54_ = 17.56 *p* < 0.001	

**Table 4:** Mean squares (MS), F and *p* values obtained from two-way ANOVA testing of the significance of the main (K—camphor or the thujone-camphor mixture, C—concentration) and interaction effects on the behavioural responses in the olfactometer (choice time) and the responses after the contact with the treated disc (escape time). Significant effects are marked in bold.

		Larval Choice Time			Adult Choice Time			Larval Escape Time		
Source of Variation	df	MS	F	*p*	MS	F	*p*	MS*100	F	*p*
Ketone (K)	1	0.6	0.10	0.753	35478	4.34	**0.042**	0.45	0.27	0.608
Concentration (C)	2	80.8	14.63	**<0.001**	21221	2.60	0.084	46.69	27.78	**<0.001**
K × C	2	0.1	0.02	0.981	433	0.05	0.948	26.87	15.99	**<0.001**
Error	54	5.5			8166			1.68		

**Table 5:** Mean squares (MS), F and *p* values obtained from three-way ANOVA testing of the significance of the main (D—developmental stage, K—camphor or thujone-camphor mixture, C—concentration) and the interaction effects on choice time in an olfactometer. Significant effects are marked in bold.

Source of Variation	df	MS	F	*p*
Dev. stage (D)	1	52.0	5.03	**0.027**
Ketone (K)	1	37.5	3.62	0.060
Concentration (C)	2	122.9	11.89	**<0.001**
D × K	1	25.7	2.48	0.118
D × C	2	3.6	0.35	0.705
K × C	2	0.4	0.04	0.964
D × K × C	2	0.04	0.003	0.997
Error	108	10.3		

## Data Availability

The data presented in this study are available on request from the corresponding author.
